# Wavelet optimal estimations for a two-dimensional continuous-discrete density function over $L^{p}$ risk

**DOI:** 10.1186/s13660-018-1868-7

**Published:** 2018-10-11

**Authors:** Lin Hu, Xiaochen Zeng, Jinru Wang

**Affiliations:** 1 Department of Basic Courses, Beijing Union University, Beijing, P.R. China; 2 Department of Applied Mathematics, Beijing University of Technology, Beijing, P.R. China

**Keywords:** Wavelets, Density estimation, Continuous-discrete density, Optimality

## Abstract

The mixed continuous-discrete density model plays an important role in reliability, finance, biostatistics, and economics. Using wavelets methods, Chesneau, Dewan, and Doosti provide upper bounds of wavelet estimations on $L^{2}$ risk for a two-dimensional continuous-discrete density function over Besov spaces $B^{s}_{r,q}$. This paper deals with $L^{p}$ ($1\leq p < \infty$) risk estimations over Besov space, which generalizes Chesneau–Dewan–Doosti’s theorems. In addition, we firstly provide a lower bound of $L^{p}$ risk. It turns out that the linear wavelet estimator attains the optimal convergence rate for $r \geq p$, and the nonlinear one offers optimal estimation up to a logarithmic factor.

## Introduction

### Introduction

The density estimation plays an important role in both statistics and econometrics. This paper considers a two-dimensional density estimation model defined over mixed continuous and discrete variables [2]. More precisely, let $(X_{1},Y_{1})$, $(X_{2},Y_{2}),\dots,(X_{n},Y_{n})$ be independent and identically distributed (i.i.d.) observations of a bivariate random variable $(X,Y)$, where *X* is a continuous random variable, and *Y* is a discrete one. The joint density function of $(X,Y)$ is given by

$$f(x,v)=\frac{\partial}{\partial x}F(x,v) $$

with $F(x,v)=P(X\leq x, Y=v)$ being the distribution function of $(X,Y)$. We are interested in estimating $f(x,v)$ from $(X_{1},Y_{1})$, $(X_{2},Y_{2}),\dots, (X_{n},Y_{n})$. This continuous-discrete density model also arises in survival analysis, economics, and social sciences. For example, consider a series system with *m* components, which fails as soon as one of the components fails. Let *X* be the failure time of the system, and let *Y* be the component whose failure resulted in the failure of the system. Then $(X,Y)$ is a bivariate continuous-discrete random variable. For more examples, see [1] and [4].

The conventional kernel method gives a nice estimation for the continuous-discrete density function [1, 10, 14]. However, it is hard to provide the optimal estimation for the densities in Besov spaces. In addition, the complexity of bandwidth selection increases the difficulty of the kernel method.

Recently, wavelet methods have made the remarkable achievements in density estimation [7, 8, 11, 12, 15] due to their time and frequency localization, multiscale decomposition, and fast algorithm in numerical computations. In fact, wavelet estimation attains optimality for densities in Besov spaces, which avoids the disadvantage of kernel methods. Using the wavelet method, Chesneau et al. [2] constructed linear and nonlinear wavelet estimators for a two-dimensional continuous-discrete density function and derived their mean integrated squared errors performance over Besov balls.

This paper addresses $L^{p}$ ($1\leq p<\infty$) risk estimations on Besov balls by using wavelet bases, which generalizes Chesneau–Dewan–Doosti’s theorems. It should be pointed out that a lower bound for $L^{p}$ risk of all estimators is derived firstly. It turns out that the linear wavelet estimator is optimal for $r\geq p$ and the nonlinear one attains optimal estimation up to a logarithmic factor.

### Notations and definitions

In this paper, we use the tensor product method to construct an orthonormal wavelet basis for $L^{2}(\mathbb{R}^{2})$, which will be used in later discussions. With a one-dimensional Daubechies scaling function $D_{2N}$ and a wavelet function $\psi_{2N}$ ($\psi_{2N}$ can be constituted from the scaling function $D_{2N}$), we construct two-dimensional tensor product wavelets *φ*, $\psi^{1}$, $\psi ^{2}$, and $\psi^{3}$ as follows:

$$\begin{gathered} \varphi(x,y):=D_{2N}(x)D_{2N}(y), \qquad \psi^{1}(x,y):=D_{2N}(x)\psi _{2N}(y), \\ \psi^{2}(x,y):=\psi_{2N}(x)D_{2N}(y), \qquad \psi^{3}(x,y):=\psi _{2N}(x)\psi_{2N}(y). \end{gathered} $$

Then *φ* and $\psi^{i}$ ($i=1, 2, 3$) are compactly supported in time domain, because Daubechies’ wavelet $D_{2N}$ and $\psi_{2N}$ are [5, 8].

Denote

$$\varphi_{j, k}(x,y):=2^{j}\varphi\bigl(2^{j}x-k_{1}, 2^{j}y-k_{2}\bigr), \qquad\psi^{i}_{j, k}(x,y):=2^{j} \psi^{i}\bigl(2^{j}x-k_{1}, 2^{j}y-k_{2} \bigr) $$

for $k=(k_{1}, k_{2})\in\mathbb{Z}^{2}$ and $i=1, 2, 3$. Then for each $f\in L^{2}(\mathbb{R}^{2})$,

$$f=\sum_{k\in\mathbb{Z}^{2}}\alpha_{j_{0},k} \varphi_{j_{0},k}+ \sum_{j=j_{0}}^{\infty}\sum _{i=1}^{3} \sum _{k\in\mathbb{Z}^{2}}\beta^{i}_{j,k} \psi_{j,k}^{i} $$

holds in $L^{2}$ sense, where $\alpha_{j,k}:=\langle f, \varphi_{j,k}\rangle$, $\beta^{i}_{j,k}:=\langle f, \psi_{j,k}^{i}\rangle$. As usual, let $P_{j}$ be the orthogonal projection operator defined by

$$P_{j}f:=\sum_{k\in\mathbb{Z}^{2}}\langle f, \varphi_{j, k}\rangle \varphi_{j, k}. $$

Details on wavelet basis can be found in [5, 8]. A scaling function *φ* is called *m*-regular if $\varphi\in C^{m}(\mathbb{R}^{2})$ and $|D^{\alpha}\varphi(x)|\leq C(1+|x|^{2})^{-l}$ for each $l\in\mathbb{Z}$ ($|\alpha|=0, 1, \ldots, m$). By the definition of tensor product wavelets we find that the scaling function *φ* is *m*-regular, since Daubechies’ function $D_{2N}$ is smooth enough for large *N*.

One of advantages of wavelet bases is that they can characterize Besov spaces, which contain Hölder spaces and $L^{2}$-Sobolev spaces as particular examples. Throughout the paper, we work within a Besov space on a compact subset of $\mathbb{R}^{2}$. The following lemma shows equivalent definitions for those spaces, which are fundamental in our discussions.

#### Lemma 1.1

([13])

*Let* *φ* *be an* *m*-*regular orthonormal scaling function with the corresponding wavelets* $\psi^{i}$ ($i=1, 2, 3$). *If* $f\in L^{r}(\mathbb{R}^{2})$, $\alpha_{j,k}=\langle f, \varphi_{j,k}\rangle$ $\beta^{i}_{j,k}=\langle f, \psi^{i}_{j,k}\rangle$, *and* $1\leq r, q\leq\infty$, $0< s< m$. *Then following assertions are equivalent*:

(i) $f\in B_{r,q}^{s}(\mathbb{R}^{2})$;
(ii) $\{2^{js}\|P_{j+1}f-P_{j}f\|_{r}\}_{j\geq0}\in l^{q}$;
(iii) $\|\{2^{j(s+1-\frac{2}{p})}\|\beta_{j,\cdot}\|_{r}\} _{j\geq0} \|_{q}< \infty$.

The Besov norm of *f* can be defined by

$$\Vert f \Vert _{B_{r,q}^{s}}:= \Vert \alpha_{j_{0},\cdot} \Vert _{r}+ \bigl\Vert \bigl\{ 2^{j(s+1-\frac{2}{p})} \Vert \beta_{j,\cdot} \Vert _{r} \bigr\} _{j\geq j_{0}} \bigr\Vert _{q}, $$

where $\|\alpha_{j_{0},\cdot}\|_{r}^{r}:=\sum_{k\in\mathbb{Z}^{2}}|\alpha _{j_{0},k}|^{r}$ and $\|\beta_{j,\cdot}\|_{r}^{r}:=\sum_{i=1}^{3} \sum_{k\in\mathbb{Z}^{2}}|\beta^{i}_{j,k}|^{r}$.

Here and further, $A\lesssim B$ means that $A\leq CB$ for some constant $C>0$ independent of *A* and *B*, $A\gtrsim B$ means $B\lesssim A$, and $A\sim B$ stands for both $A\lesssim B$ and $A\gtrsim B$.

#### Remark 1.1

By (i) and (ii) of Lemma 1.1 we observe that

$$\Vert P_{j}f-f \Vert _{r}= \Biggl\Vert \sum _{l=j}^{\infty}(P_{l+1}f-P_{l} f) \Biggr\Vert _{r}\leq\sum_{l=j}^{\infty} \Vert P_{l+1}f-P_{l}f \Vert _{r}\lesssim\sum _{l=j}^{\infty}2^{-ls} \lesssim2^{-js} $$

for $f\in B_{r,q}^{s}(\mathbb{R}^{2})$. Hence

1.1 $$\begin{aligned} \Vert P_{j}f-f \Vert _{r}\lesssim2^{-js}. \end{aligned}$$

#### Remark 1.2

When $r\leq p$, Lemma 1.1(i) and (iii) imply that, for $s'-\frac{2}{p}=s-\frac{2}{r}>0$,

$$B_{r,q}^{s}\bigl(\mathbb{R}^{2}\bigr) \hookrightarrow B_{p,q}^{s'}\bigl(\mathbb{R}^{2} \bigr), $$

where $A\hookrightarrow B$ stands for a Banach space *A* continuously embedded in another Banach space *B*. More precisely, $\|u\|_{B}\leq C\| u\|_{A}$ ($u\in A$) for some constant $C>0$.

#### Lemma 1.2

([13])

*Let* $\varphi\in L^{2}(\mathbb{R}^{2})$ *be a scaling function or a wavelet with* $\sup_{k\in\mathbb {Z}^{2}}|\varphi(x-k)|< \infty$. Then, for $\lambda=\{\lambda_{k}\}\in l^{p}(\mathbb{Z}^{2})$ and $1\leq p\leq\infty$,

$$\biggl\Vert \sum_{k\in\mathbb{Z}^{2}}\lambda_{k} \varphi_{j,k} \biggr\Vert _{p}\sim 2^{j(1-2/p)} \Vert \lambda \Vert _{p}. $$

Here $\|\lambda\|_{p}$ is the $l^{p}(\mathbb{Z}^{2})$ norm of $\lambda\in l^{p}(\mathbb{Z}^{2})$:

$$\Vert \lambda \Vert _{p}:= \textstyle\begin{cases} (\sum_{k\in\mathbb{Z}^{2}} \vert \lambda_{k} \vert ^{p})^{1/p} & \mbox{if } p< \infty ,\\ \sup_{k\in\mathbb{Z}^{2}} \vert \lambda_{k} \vert & \mbox{if } q=\infty. \end{cases} $$

### Main results

In this subsection, we state our main results and discuss relations to some other work. To do that, we propose a new bivariate function $f_{\ast}(x, y)$, which is an improved one of that in [2]. Define

$$f_{\ast}(x,y):=\sum_{v=1}^{m}u(y,v)P(Y=v)f(x|Y=v) $$

with

$$u(y,v)= \textstyle\begin{cases} \frac{1}{1+e^{\frac{1}{y-v}+\frac{1}{y-v+1}}}1_{(v-1,v)}(y)+ \frac{e^{\frac{1}{y-v}+\frac{1}{y-v-1}}}{1+e^{\frac{1}{y-v}+\frac {1}{y-v-1}}}1_{(v,v+1)}(y), & y\neq v,\\ 1, & y=v, \end{cases} $$

where ${1}_{D}$ is the indicator function of a set *D*.

The construction of $f_{\ast}$ follows the idea proposed by Chesneau [2] but is different from [2]. The weight $u(y,v)$ equals to characteristic function $1_{\{v-\frac{1}{2}\leq y< v+\frac{1}{2}\}}$ in [2]. By a careful verification our weight $u(y,v)$ is differentiable with respect to *y* for each $v\in\{1, 2, \ldots, m\}$. The modification of $u(y,v)$ from the characteristic function to the smooth one makes $f_{\ast}$ continuous in *y*. It is easy to see that, for any $y=v\in\{1, 2, \ldots, m\}$,

$$f_{\ast}(x,y)=f(x,v). $$

Hence, the problem is converted to construct an estimator of $f_{\ast}$. As in [2], we assume that $f_{\ast}$ belongs to the space $B_{r,q}^{s}(H, Q)$ or, equivalently, $f_{\ast}$ belongs to the Besov ball

$$B_{r,q}^{s}(H):=\bigl\{ f, f\in B_{r,q}^{s} \bigl(\mathbb{R}^{2}\bigr) \mbox{ and } \Vert f \Vert _{B_{r,q}^{s}}\leq H\bigr\} $$

and that the support of $f_{\ast}(x, \cdot)$ is contained in $[-Q, Q]$ for fixed *v* ($Q>0$, $v=1, 2, \ldots, m$).

To introduce the wavelet estimator, we need the wavelet coefficient estimators of $\alpha_{j,k}$ and $\beta_{j,k}^{i}$:

1.2 $$\begin{aligned} \hat{\alpha}_{j,k}=\frac{1}{n}\sum_{l=1}^{n} \int_{\mathbb{R}} \varphi_{j,k}(X_{l},y)u(y, Y_{l})\,dy, \qquad\hat{\beta}^{i}_{j,k}= \frac {1}{n}\sum_{l=1}^{n} \int_{\mathbb{R}} \psi_{j,k}^{i}(X_{l},y)u(y, Y_{l})\,dy. \end{aligned}$$

Define $\wedge_{j_{0}}:=\{k\in\mathbb{Z}^{2}, \operatorname{supp} f_{\ast}\cap \operatorname{supp} \varphi_{j_{0},k} \neq\emptyset\}$. When $f_{\ast}$ and *φ* have compact supports, the cardinality of $\wedge_{j}$ satisfies $\sharp\wedge_{j}\lesssim2^{2j}$. Then the linear wavelet estimator of $f_{\ast}$ is given as follows:

1.3 $$\begin{aligned} \hat{f}^{{\mathrm{lin}}}_{n}(x,y):=\sum_{k\in\wedge _{j_{0}}} \hat{\alpha}_{j_{0}, k}\varphi_{j_{0}, k}(x,y), \end{aligned}$$

where $j_{0}$ is chosen such that $2^{j_{0}}\sim n^{\frac{1}{2s'+1}}$, $s':=s-(\frac{2}{r}-\frac{2}{p})_{+}$, and $x_{+}:=\max\{x,0\}$.

To obtain a nonlinear estimator, we take $j_{0}$ and $j_{1}$ such that $2^{j_{1}}\sim \frac{n}{\ln n}$ and $2^{j_{0}}\sim n^{\frac{1}{2m+1}}$ with $m>s$. Define $\wedge_{j}:=\{k\in\mathbb{Z}^{2}, \operatorname{supp} f_{\ast}\cap \operatorname{supp} \psi^{i}_{j, k} \neq\emptyset\}$ and $\lambda_{j}:=\frac{T}{2}2^{-\frac{j}{2}}\sqrt{\frac{\ln n}{n}}$ (*T* is the constant described as Lemma 2.3). Then the nonlinear estimator is given by

1.4 $$\begin{aligned} \hat{f}^{{\mathrm{non}}}_{n}(x,y):=\sum_{k\in\wedge _{j_{0}}} \hat{\alpha}_{j_{0}, k}\varphi_{j_{0},k}(x,y)+\sum _{j=j_{0}}^{j_{1}}\sum_{i=1}^{3} \sum_{k\in \wedge_{j}}\hat{\beta}^{i}_{j,k}1_{\{|\hat{\beta}^{i}_{j,k}|>\lambda _{j}\}} \psi_{j, k}^{i}(x,y). \end{aligned}$$

From the definition of $\hat{f}^{{\mathrm{non}}}_{n}$ we find that the nonlinear estimator has the advantage to be adaptive, since it does not depend on the indices *s*, *r*, *q* and *H* in its construction.

The following theorem gives a lower bound estimation for $L^{p}$ risk.

#### Theorem 1.1

*Let* *f̂* *be an estimator of* $f_{\ast}\in B_{r,q}^{s}(H)$ *with* $s>\frac{2}{r}$ *and* *r*, $q\geq1$. *Then there exists* $C>0$ *such that*, *for* $1\leq p<\infty$,

$$\sup_{f_{\ast}\in B_{r,q}^{s}(H)} E \Vert \hat{f}_{n}-f_{\ast} \Vert _{p}^{p}\geq C \max\biggl\{ n^{-\frac{sp}{2s+1}}, \biggl( \frac{\ln n}{n}\biggr)^{-\frac{(s-\frac {2}{r}+\frac{2}{p})p}{2(s-\frac{2}{r})+1}} \biggr\} . $$

The upper bounds of the linear and nonlinear wavelet estimators are provided by Theorems 1.2 and 1.3, respectively.

#### Theorem 1.2

*Let* $\hat{f}^{{\mathrm{lin}}}_{n}$ *be the estimator of* $f_{\ast}\in B_{r,q}^{s}(H, Q)$ *defined by* (1.3) *with* $1\leq r,q<\infty$, $s>0$. *If the density of* *X* *is bounded*, *then for* $r\geq p\geq1$ *or* $r\leq p<\infty$ *and* $s>\frac{2}{r}$,

$$\sup_{f_{\ast}\in B_{r,q}^{s}(H, Q)} E \bigl\Vert \hat{f}^{{\mathrm{lin}}}_{n}-f_{\ast} \bigr\Vert _{p}^{p}\lesssim n^{-\frac {ps'}{2s'+1}} $$

*with* $s'=s-(\frac{2}{r}-\frac{2}{p})_{+}$ *and* $x_{+}:=\max(x,0)$.

#### Remark 1.3

If $r\geq2$, $p=2$ and $s>0$, $s'=s$, then Theorem 1.2 reduces to Theorem 4.1 in [2]. In addition, Theorem 1.2 does not make any restriction on *Q*, and so the assumptions are weaker than in [2]. Theorem 1.2 extends the corresponding theorem of [2] from $p=2$ to $p\in[1, \infty)$.

When $r\geq p$, $s'=s$ and the linear wavelet estimator $\hat{f}^{{\mathrm{lin}}}_{n}$ attains optimality thanks to Theorems 1.1 and 1.2. However, the linear estimator does not offer optimal estimation for $r< p$, because of $s'< s$ and $\frac{s'}{2s'+1}<\frac{s}{2s+1}$ in this case.

To give a suboptimal estimation for $r< p$, we need the nonlinear wavelet estimators defined by (1.4).

#### Theorem 1.3

*Let* $\hat{f}^{{\mathrm{non}}}_{n}$ *be the estimator of* $f_{\ast}\in B_{r,q}^{s}(H, Q)$ *defined by* (1.4) *with* $1\leq r,q<\infty$, $s>0$. *If the density of* *X* *is bounded*, *then for* $r\geq p\geq1$ *or* $r\leq p<\infty$ *and* $s>\frac{2}{r}$,

$$\sup_{f_{\ast}\in B_{r,q}^{s}(H, Q)} E \bigl\Vert \hat{f}^{{\mathrm {non}}}_{n}-f_{\ast} \bigr\Vert _{p}^{p}\lesssim (\ln n)^{p}\biggl( \frac{\ln n}{n}\biggr)^{\alpha p} $$

*with* $\alpha:=\min \{\frac{s}{2s+1}, \frac{s-\frac{2}{r}+\frac{2}{p}}{2(s-\frac {2}{r})+1}\}$.

#### Remark 1.4

Theorems 1.1 and 1.3 tell us that the nonlinear estimator is suboptimal up to a logarithmic factor. Moreover, if $p=2$ and $\{r\geq2, s>0\}$ or $\{1\leq r<2, s>\frac{2}{r}\}$, then $\alpha=\frac{s}{2s+1}$, and Theorem 1.3 is the same as Theorem 4.2 in [2] up to a logarithmic factor. Hence Theorem 1.3 can be considered as an extension of Theorem 4.2 in [2] from $p=2$ to $p\in[1, \infty)$.

In particular, we can extend the theorems to the multidimensional case as in [3] by using the technique developed by [9]. It is a challenging problem to study the estimation of a multivariate continuous-discrete conditional density. We refer to [3] for further details.

## Some lemmas

We shall show several lemmas in this section, which are needed for proofs of our main theorems.

### Lemma 2.1

*Let* $\hat{\alpha}_{j,k}$ *and* $\hat{\beta}_{j,k}$ *be defined by* (1.2). *Then*

$$E (\hat{\alpha}_{j,k})=\alpha_{j,k} \quad \textit{and} \quad E \bigl(\hat {\beta}^{i}_{j,k}\bigr)=\beta^{i}_{j,k} $$

*for* $j\geq j_{0}$, $k\in\mathbb{Z}^{2}$, *and* $i=1, 2, 3$.

### Proof

Denote $c_{j,k}(v)=\int\phi_{j,k_{2}}(y)u(y,v)\,dy$. Then

$$\hat{\alpha}_{j,k}=\frac{1}{n}\sum_{i=1}^{n} \int\varphi _{j,k}(X_{i},y)u(y, Y_{i})\,dy= \frac{1}{n}\sum_{i=1}^{n}\phi _{j,k_{1}}(X_{i})c_{j,k}(Y_{i}). $$

Since $(X_{1},Y_{1})$, $(X_{2},Y_{2}), \dots, (X_{n},Y_{n})$ are independent and identically distributed, we have

$$\begin{aligned} E(\hat{\alpha}_{j,k})&=E\bigl( \phi_{j,k_{1}}(X_{1})c_{j,k}(Y_{1})\bigr)=E \bigl(E\bigl(\phi _{j,k_{1}}(X_{1})c_{j,k}(Y_{1})|Y_{1} \bigr)\bigr) \\ & =E\bigl(c_{j,k}(Y_{1})E\bigl(\phi_{j,k_{1}}(X_{1})|Y_{1} \bigr)\bigr)=E\biggl(c_{j,k}(Y_{1}) \int \phi_{j,k_{1}}(x)f(x|Y_{1})\,dx\biggr) \\ & =\sum_{v=1}^{m}P(Y_{1}=v)c_{j,k}(v) \int\phi _{j,k_{1}}(x)f(x|Y_{1}=v)\,dx \\ & = \int \int\Biggl(\sum_{v=1}^{m}P(Y_{1}=v)u(y,v)f(x|Y_{1}=v) \Biggr)\phi _{j,k_{1}}(x)\phi_{j,k_{2}}(y) \,dx \,dy \\ & = \int \int f_{\ast}(x,y)\varphi_{j,k}(x, y) \,dx \,dy= \alpha_{j,k}. \end{aligned} $$

Similarly to the previous arguments, $E (\hat{\beta}^{i}_{j,k})=\beta ^{i}_{j,k}$. The proof of Lemma 2.1 is done. □

To show Lemma 2.2, we introduce Rosenthal’s inequality.

### Rosenthal’s inequality

([8])

Let $X_{1}, X_{2}, \ldots, X_{n}$ be independent random variables such that $EX_{l} =0$ and $E |X_{l} |^{p}<\infty$ ($l=1,2,\ldots, n$). Then, with $C_{p}>0$,

$$E \Biggl\vert \sum_{l=1}^{n}X_{l} \Biggr\vert ^{p}\leq \textstyle\begin{cases} C_{p} [\sum_{l=1}^{n}E \vert X_{l} \vert ^{p}+ (\sum_{l=1}^{n}E \vert X_{l} \vert ^{2} )^{p/2} ] , & p\geq2,\\ C_{p} (\sum_{l=1}^{n}E \vert X_{l} \vert ^{2} )^{p/2}, & 0< p\leq 2. \end{cases} $$

### Lemma 2.2

*Let* $\hat{\alpha}_{j,k}$ *and* $\hat{\beta}_{j,k}$ *be defined by* (1.2). *If the density of* *X* *is bounded*, *then there exists a constant* $C>0$ *such that*

$$E \vert \hat{\alpha}_{j,k}-\alpha_{j,k} \vert ^{p}\leq2^{-\frac{p}{2}j}n^{-\frac {p}{2}} \quad\textit{and} \quad E \vert \hat{\beta}_{j,k}-\beta_{j,k} \vert ^{p} \leq2^{-\frac{p}{2}j}n^{-\frac{p}{2}} $$

*for* $1\leq p<\infty$ *and* $2^{j}\leq n$.

### Proof

We only prove the first inequality, since the second one is similar. By the definition of $\hat{\alpha}_{j,k}$,

$$\hat{\alpha}_{j,k}=\frac{1}{n}\sum_{l=1}^{n} \int_{\mathbb{R}} \varphi_{j,k}(X_{l}, y)u(y, Y_{l})\,dy=\frac{1}{n}\sum_{l=1}^{n} \phi_{j,k_{1}}(X_{l})c_{j,k_{2}}(Y_{l}), $$

where $c_{j,k_{2}}(Y_{l}):=\int_{\mathbb{R}} \phi_{j,k_{2}}(y)u(y,Y_{l})\,dy$, and *ϕ* is a one-dimensional Daubechies scaling function $D_{2N}$. Since $|u(y,v)|\leq2$, we obtain that

2.1 $$\begin{aligned} \bigl\vert c_{j,k_{2}}(Y_{l}) \bigr\vert \leq \int_{\mathbb{R}} \bigl\vert \phi _{j,k_{2}}(y) \bigr\vert \bigl\vert u(y,Y_{l}) \bigr\vert \,dy\leq 2^{-\frac{j}{2}} \Vert \phi \Vert _{1} \end{aligned}$$

and

2.2 $$\begin{aligned} E \bigl\vert \phi_{j,k_{1}}(X_{l})c_{j,k_{2}}(Y_{l}) \bigr\vert ^{p}\lesssim 2^{-\frac{p}{2}j}E \bigl\vert \phi_{j,k_{1}}(X_{l}) \bigr\vert ^{p} \lesssim2^{-\frac{p}{2}j} \int_{\mathbb{R}} \bigl\vert \phi _{j,k_{1}}(x) \bigr\vert ^{p}f_{X}(x)\,dx\lesssim2^{-j} \end{aligned}$$

due to the boundedness of $f_{X}$. Define $\xi_{l}:=\phi _{j,k_{1}}(X_{l})c_{j,k_{2}}(Y_{l})-\alpha_{j,k}$. Then

2.3 $$\begin{aligned} E \vert \xi_{l} \vert ^{p}= E \bigl\vert \phi _{j,k_{1}}(X_{l})c_{j,k_{2}}(Y_{l})- \alpha_{j,k} \bigr\vert ^{p} \lesssim E \bigl\vert \phi_{j,k_{1}}(X_{l})c_{j,k_{2}}(Y_{l}) \bigr\vert ^{p}+E \vert \alpha_{j,k} \vert ^{p}. \end{aligned}$$

It follows from Lemma 2.1 and Jensen’s inequality that

$$E \vert \alpha_{j,k} \vert ^{p}= \bigl\vert E \bigl[ \phi _{j,k_{1}}(X_{l})c_{j,k_{2}}(Y_{l}) \bigr] \bigr\vert ^{p}\leq E \bigl\vert \phi _{j,k_{1}}(X_{l})c_{j,k_{2}}(Y_{l}) \bigr\vert ^{p}. $$

Hence (2.3) reduces to

2.4 $$\begin{aligned} E \vert \xi_{l} \vert ^{p}\lesssim E \bigl\vert \phi _{j,k_{1}}(X_{l})c_{j,k_{2}}(Y_{l}) \bigr\vert ^{p}\lesssim2^{-j} \end{aligned}$$

thanks to (2.2). By the definition of $\hat{\alpha}_{j,k}$ and $\xi_{l}$, $\hat{\alpha }_{j,k}-\alpha_{j,k}=\frac{1}{n}\sum_{l=1}^{n}\xi_{l}$, where $\xi_{1}, \xi_{2}, \ldots, \xi_{n}$ are independent because $(X_{1}, Y_{1})$, $(X_{2}, Y_{2}), \ldots, (X_{n}, Y_{n})$ also are. On the other hand, Lemma 2.1 implies $E(\xi_{l})=0$. Then Rosenthal inequality leads to

2.5 $$\begin{aligned} E \vert \hat{\alpha}_{j,k}-\alpha_{j,k} \vert ^{p}=E \Biggl\vert \frac{1}{n} \sum _{l=1}^{n}\xi_{l} \Biggr\vert ^{p}\lesssim \textstyle\begin{cases} n^{-p} [\sum_{l=1}^{n}E \vert \xi_{l} \vert ^{p}+ (\sum_{l=1}^{n}E \vert \xi_{l} \vert ^{2} )^{\frac{p}{2}} ], & p\geq 2,\\ n^{-p} (\sum_{l=1}^{n}E \vert \xi_{l} \vert ^{2} )^{\frac{p}{2}}, & 1\leq p\leq2. \end{cases}\displaystyle \end{aligned}$$

By (2.4) we know that

$$n^{-p} \Biggl(\sum_{l=1}^{n}E \vert \xi_{l} \vert ^{2} \Biggr)^{\frac{p}{2}}\lesssim n^{-p}\bigl(n2^{-j}\bigr)^{\frac {p}{2}}\lesssim n^{-\frac{p}{2}}2^{-\frac{p}{2}j} $$

for $1\leq p<2$ and

$$n^{-p} \Biggl[\sum_{l=1}^{n}E \vert \xi_{l} \vert ^{p}+ \Biggl(\sum _{l=1}^{n}E \vert \xi_{l} \vert ^{2} \Biggr)^{\frac{p}{2}} \Biggr] \lesssim n^{-p} \bigl(n2^{-j}+n^{\frac{p}{2}}2^{-\frac{p}{2}j}\bigr)\lesssim n^{-\frac{p}{2}}2^{-\frac{p}{2}j} $$

for $p\geq2$ thanks to the assumption $2^{j}\leq n$. Combining these with (2.5), we receive the desired conclusion

$$E \vert \hat{\alpha}_{j,k}-\alpha_{j,k} \vert ^{p}\lesssim2^{-\frac {p}{2}j}n^{-\frac{p}{2}}. $$

This completes the proof. □

To prove Lemma 2.3, we need the well-known Bernstein inequality.

### Bernstein’s inequality

([8])

Let $X_{1}, X_{2}, \ldots, X_{n}$ be i.i.d. random variables with $E(X_{i})=0$ and $\|X_{i}\|_{\infty }\leq M$. Then, for each $\gamma>0$,

$$P \Biggl\{ \Biggl\vert \frac{1}{n}\sum_{i=1}^{n}X_{i} \Biggr\vert >\gamma \Biggr\} \leq2\exp \biggl(-\frac{n\gamma^{2}}{2[E(X_{i}^{2})+ \Vert X \Vert _{\infty }\gamma/3]} \biggr). $$

The next lemma is an extension of Proposition 4.2 in [2].

### Lemma 2.3

*Let* $2^{j}\leq\frac{n}{\ln n}$, $\hat{\beta}_{j,k}^{i}$ ($i=1,2,3$) *be defined in* (1.2). *If the density of* *X* *is bounded*, *then for each* $\varepsilon>0$, *there exists* $T>0$ *such that*, *for* $j\geq0$ *and* $k\in\mathbb{Z}^{2}$,

2.6 $$\begin{aligned} P \biggl\{ \bigl\vert \hat{\beta}^{i}_{j,k}- \beta^{i}_{j,k} \bigr\vert >\frac {T}{2}2^{-\frac{1}{2}j} \sqrt{\frac{\ln n}{n}} \biggr\} \lesssim2^{-\varepsilon j}. \end{aligned}$$

### Proof

We only show (2.6) for $i=1$. By the definition of $\hat{\beta}^{1}_{j,k}$, $\hat{\beta}^{1}_{j,k}=\frac{1}{n}\sum_{l=1}^{n}\int_{\mathbb{R}} \psi ^{1}_{j,k}(X_{l}, y)u(y, Y_{l})\,dy$, and

$$\hat{\beta}^{1}_{j,k}-\beta^{1}_{j,k}= \frac{1}{n}\sum_{l=1}^{n} \bigl[\phi _{j,k_{1}} (X_{l})d_{j,k_{2}}(Y_{l})- \beta^{1}_{j,k} \bigr], $$

where $d_{j,k_{2}}(Y_{l}):=\int_{\mathbb{R}} \psi_{j,k_{2}}(y)u(y, Y_{l})\,dy$ (*ϕ*, *ψ* stand for the one-dimensional Daubechies scaling function and wavelet function, respectively). Define $\eta_{l}:=\phi_{j,k_{1}}(X_{l})d_{j,k_{2}}(Y_{l})-\beta^{1}_{j,k}$. Then $\hat{\beta}^{1}_{j,k}-\beta^{1}_{j,k}=\frac{1}{n}\sum_{l=1}^{n}\eta_{l}$ and $E(\eta_{l})=0$ because of $\beta^{l}_{j,k}=E(\hat{\beta}^{l}_{j,k})= E [\phi_{j,k_{1}}(X_{i})d_{j,k_{2}}(Y_{i}) ]$.

Using (2.1) with *ψ* instead of *ϕ*, we get $|d_{j,k_{2}}(Y_{l}) |\lesssim2^{-\frac{j}{2}}$. Note that $|\phi_{j,k_{1}}(X_{l})|:=2^{\frac{j}{2}} |\phi(2^{j}X_{l}-k_{1})|\leq2^{\frac{j}{2}}\|\phi\|_{\infty}$. Then $|\phi_{j,k_{1}}(X_{l})d_{j,k_{2}}(Y_{l}) |\lesssim1$ and $|\beta^{1}_{j,k}|= |E [\phi _{j,k_{1}}(X_{l})d_{j,k_{2}}(Y_{l}) ] |\lesssim1$. Hence

2.7 $$\begin{aligned} \vert \eta_{l} \vert \leq \bigl\vert \phi_{j,k_{1}}(X_{l}) d_{j,k_{2}}(Y_{l})-\beta^{1}_{j,k} \bigr\vert \lesssim1. \end{aligned}$$

By replacing $c_{j,k_{2}}$ and $\alpha_{j,k}$ with $d_{j,k_{2}}$ and $\beta^{1}_{j,k}$, respectively, arguments similar to (2.1)–(2.4) show that

2.8 $$\begin{aligned} E \vert \eta_{l} \vert ^{p}\lesssim2^{-j}. \end{aligned}$$

Because $\eta_{1}, \eta_{2}, \ldots, \eta_{n}$ are i.i.d. and $E(\eta_{l})=0$ ($l=1, 2, \ldots, n$), Bernstein’s inequality tells us that

2.9 $$\begin{aligned} P \Biggl\{ \bigl\vert \hat{\beta}^{l}_{j,k}- \beta^{l}_{j,k} \bigr\vert = \Biggl\vert \frac{1}{n}\sum_{l=1}^{n} \eta_{l} \Biggr\vert >\frac{T}{2}2^{-\frac{1}{2}j} \sqrt{ \frac{\ln n}{n}} \Biggr\} \leq2\exp \biggl(-\frac{n\lambda_{j}^{2}}{ 2[E(\eta_{l}^{2})+\frac{\lambda_{j}}{3} \Vert \eta \Vert _{\infty}]} \biggr) \end{aligned}$$

with $\lambda_{j}=\frac{T}{2}2^{-\frac{1}{2}j}\sqrt{\frac{\ln n}{n}}$. This with (2.7)–(2.8) implies

$$\frac{n\lambda_{j}^{2}}{2[E(\eta_{l}^{2})+\frac{\lambda_{j}}{3} \Vert \eta \Vert _{\infty}]} \geq \frac{T^{2}\ln n}{8 (C_{1}+\frac{C_{2}}{6}T 2^{\frac{j}{2}}\sqrt{\frac{\ln n}{n}} )}\geq \frac{T^{2}\ln n}{8 (C_{1}+\frac{C_{2}}{6}T )} $$

because $2^{\frac{j}{2}}\sqrt{\frac{\ln n}{n}}\leq1$ by the assumption $2^{j}\leq\frac{n}{\ln n}$. Note that $\ln n> j\ln2$ due to $n\geq2^{j}\ln n>2^{j}$. Hence

$$\frac{n\lambda_{j}^{2}}{2[E(\eta_{l}^{2})+\frac{\lambda_{j}}{3} \Vert \eta \Vert _{\infty}]} \geq\frac{T^{2}\ln2}{8 (C_{1}+\frac{C_{2}}{6}T )}j>\varepsilon j $$

by choosing $T>0$ such that $\frac{T^{2}\ln2}{8 (C_{1}+\frac {C_{2}}{6}T )}>\varepsilon$. Then (2.9) reduces to

$$P \biggl\{ \bigl\vert \hat{\beta}^{1}_{j,k}- \beta^{1}_{j,k} \bigr\vert >\frac{T}{2}2^{-\frac{1}{2}j} \sqrt{\frac{\ln n}{n}} \biggr\} \leq2^{-\varepsilon j}, $$

which concludes (2.6) with $i=1$. Similarly, the conclusions with $i=2,3$ hold. This completes the proof. □

At the end of this section, we introduce two classical lemmas, which are needed for the proof of lower bound.

### Lemma 2.4

(Varshamov–Gilbert lemma, [11])

*Let* $\Theta:= \{\varepsilon=(\varepsilon_{1}, \varepsilon_{2}, \ldots, \varepsilon_{m}) , \varepsilon_{i}\in\{0, 1\} \}$. *Then there exists a subset* $(\varepsilon^{0}, \varepsilon^{1}, \ldots, \varepsilon^{T})$ *of* Θ *with* $\varepsilon^{0}=(0, 0, \ldots, 0)$ *such that* $T\geq2^{\frac{m}{8}}$ *and*

$$\sum_{k=1}^{m} \bigl\vert \varepsilon_{k}^{i}-\varepsilon_{k}^{j} \bigr\vert \geq \frac{m}{8} \quad (0\leq i\neq j\leq T). $$

*To state Fano’s lemma*, *we introduce a concept*: *When* *P* *is absolutely continuous with respect to* *Q* (*denoted by* $P\ll Q$), *the Kullback divergence of* *P* *and* *Q* *between two measures* *P* *and* *Q* *is defined by*

$$K(P, Q):= \int p(x)\ln\frac{p(x)}{q(x)}\,dx, $$

*where* $p(x)$ *and* $q(x)$ *are the density functions of* *P* *and* *Q*, *respectively*.

### Lemma 2.5

(Fano’s lemma, [6])

*Let* $(\Omega, \mathcal{F}, P_{k})$ *be a probability spaces*, *and let* $A_{k}\in\mathcal{F}$, $k=0, 1, \ldots , m$. *If* $A_{k}\cap A_{k'}=\emptyset$ *for* $k\neq k'$, *then with* $A^{C}$ *standing for the complement of* *A* *and* $\mathcal{K}_{m}:=\inf_{0\leq k\leq m}\frac{1}{m}\sum_{k\neq k'}K(P_{k}, P_{k'})$,

$$\sup_{0\leq k\leq m}P_{k}\bigl(A_{k}^{C} \bigr)\geq\min\biggl\{ \frac{1}{2}, \sqrt{m} \exp \bigl(-3e^{-1}- \mathcal{K}_{m}\bigr)\biggr\} , $$

*where* $K(P_{k}, P_{k'})$ *is the Kullback distance of* $P_{k}$ *and* $P_{k'}$ ($k=0, 1, \ldots, m$).

## Proofs of lower bounds

We rewrite Theorem 1.1 as follows before giving its proof.

### Theorem 3.1

*Let* $\hat{f}_{n}$ *be an estimator of* $f_{\ast}\in B_{r,q}^{s}(H)$ *with* $s>\frac{2}{r}$ *and* $1\leq r,q\leq \infty$. *Then*, *for* $1\leq p<\infty$,

$$\sup_{f_{\ast}\in B_{r,q}^{s}(H)} E \Vert \hat{f}_{n}-f_{\ast} \Vert _{p}^{p}\gtrsim\max \biggl\{ n^{-\frac{sp}{2s+1}}, \biggl( \frac{\ln n}{n} \biggr)^{\frac{(s-\frac{2}{r}+\frac{2}{p})p}{2(s-\frac{2}{r})+1}} \biggr\} . $$

### Proof

As in Sect. 1, we take the two-dimensional tensor product wavelet

$$\psi^{1}(x, y):=D_{2N}(x)\psi_{2N}(y), $$

where $D_{2N}(\cdot)$ and $\psi_{2N}(\cdot)$ are the one-dimensional Daubechies scaling function and wavelet function, respectively. Then $\psi^{1}$ is *m*-regular ($m>s$) for large *N*, and

$$\operatorname{supp} \psi^{1}\subseteq[0,2N-1]\times[-N+1,N] $$

due to $\operatorname{supp} D_{2N}\subseteq[0,2N-1]$ and $\operatorname{supp} \psi_{2N}\subseteq[-N+1,N]$. Then there exists a compactly supported density function $g_{0}$ such that $\int_{\mathbb{R}^{2}} g_{0}(x)\,dx=1$, $g_{0}(x)|_{[0,2N-1]\times[-N+1,N]}=c_{0}$, and $g_{0}\in B_{r,q}^{s}(H)$. Define $\Delta_{j}:=\Delta_{j}^{1}\times\Delta_{j}^{2}$ with

$$\Delta_{j}^{1}:=\bigl\{ 0, 2N, 4N, \ldots, 2 \bigl(2^{j}-1\bigr)N\bigr\} , \qquad\Delta _{j}^{2}:= \bigl\{ 0, \pm2N, \pm4N, \ldots, \pm2\bigl(2^{j-1}-1\bigr)N\bigr\} . $$

Then $\sharp\Delta_{j}=2^{j}(2^{j}-1)\sim2^{2j}$ ($\sharp\Delta_{j}$ denotes the cardinality of $\Delta_{j}$). Denote $a_{j}:=2^{-(2s+1)j}$ and

$$\wedge:= \biggl\{ g_{\varepsilon}(x, y)=g_{0}(x, y)+a_{j} \sum_{k\in\Delta_{j}}\varepsilon_{k} \psi^{1}_{j,k}(x, y), \varepsilon_{k}\in\{0, 1\} \biggr\} . $$

Obviously, the supports of $\psi^{1}_{j,k}$ and $\psi^{1}_{j,k'}$ are disjoint for $k\neq k'\in\Delta_{j}$ and $\operatorname{supp} \psi^{1}_{j,k}\subseteq \operatorname{supp} g_{0}$. When $(x, y)\in[0,2N-1]\times[-N+1,N]$,

$$g_{\varepsilon}\geq c_{0}-a_{j} \bigl\Vert \psi^{1}_{j,k} \bigr\Vert _{\infty}\geq c_{0}-2^{-2sj} \bigl\Vert \psi^{1} \bigr\Vert _{\infty}>0 $$

for large *j*. On the other hand,

$$\int_{\mathbb{R}^{2}} g_{\varepsilon}(x, y)\,dx= \int_{\mathbb{R}^{2}} g_{0}(x, y)\,dx=1. $$

Hence $g_{\varepsilon}$ is a bivariate density function for $\varepsilon=(\varepsilon_{k})_{k\in\Delta_{j}}$.

Moreover, $g_{\varepsilon}\in B^{s}_{r,q}(H)$. In fact, for $\varepsilon_{k}\in\{0, 1\}$, $\sum_{k\in\Delta_{j}} |\varepsilon _{k}|^{r}\leq2^{2j}$ and

$$2^{j(s+1-\frac{2}{r})}a_{j} \biggl(\sum_{ k\in\Delta_{j}} \vert \varepsilon _{k} \vert ^{r} \biggr)^{\frac{1}{r}} \leq1. $$

By Lemma 1.1, $\|a_{j}\sum_{k\in\Delta_{j}}\varepsilon_{k}\psi^{1}_{j,k}\| _{B^{s}_{r,q}}\leq H$. This with $g_{0}\in B_{r,q}^{s}(H)$ implies $g_{\varepsilon}\in B^{s}_{r,q}(H)$.

According to Lemma 2.4 (Varshamov–Gilbert theorem), for $\Omega= \{\varepsilon=(\varepsilon_{k})_{k\in\Delta_{j}}, \varepsilon_{k}\in\{0, 1\} \}$, there exists a subset $\{\varepsilon^{(0)}, \varepsilon ^{(1)},\ldots,\varepsilon^{(M)} \}$ of Ω such that $M\geq2^{\frac{2^{2j}}{8}}$, $\varepsilon^{(0)}=(0,0,\ldots, 0)$, and for $m,n=0,1,\ldots,M$, $m\neq n$,

3.1 $$\begin{aligned} \sum_{k\in\Delta_{j}} \bigl\vert \varepsilon _{k}^{(m)}-\varepsilon_{k}^{(n)} \bigr\vert \geq\frac{2^{2j}}{8}. \end{aligned}$$

Denote $\wedge':= \{ g_{\varepsilon^{(0)}}, g_{\varepsilon^{(1)}}, \ldots, g_{\varepsilon^{(M)}} \}$. Then $\wedge'\subseteq\wedge$, and for $g_{\varepsilon^{(m)}}, g_{\varepsilon^{(n)}}\in\wedge'$,

$$\Vert g_{\varepsilon^{(m)}}-g_{\varepsilon^{(n)}} \Vert _{p}^{p}=a_{j}^{p} \sum_{k\in\Delta_{j}} \bigl\vert \varepsilon_{k}^{m}- \varepsilon_{k}^{n} \bigr\vert ^{p} \bigl\Vert \psi^{1}_{j,k} \bigr\Vert _{p}^{p}= 2^{-2(sp+1)j} \bigl\Vert \psi^{1} \bigr\Vert _{p}^{p}\sum_{k\in\Delta_{j}} \bigl\vert \varepsilon _{k}^{m}-\varepsilon_{k}^{n} \bigr\vert ^{p}, $$

since the supports of $\psi^{1}_{j,k}$ ($k\in\Delta_{j}$) are mutually disjoint. This with (3.1) leads to

$$\Vert g_{\varepsilon^{(m)}}-g_{\varepsilon^{(n)}} \Vert _{p}^{p} \geq C_{1}2^{-2psj}:=\delta_{j}^{p}. $$

Define

$$A_{\varepsilon^{(i)}}:= \biggl\{ \Vert \hat{f}_{n}-g_{\varepsilon^{(i)}} \Vert _{p}< \frac{\delta_{j}}{2} \biggr\} , $$

$i=0, 1, 2, \ldots, M$. Then $A_{\varepsilon^{(m)}}\cap A_{\varepsilon^{(n)}}=\emptyset$ for $m\neq n$. Denote by $P^{n}_{f}$ the probability measure with the density $f^{n}(x,y):=\prod_{i=1}^{n}f(x_{i},y_{i})$. By the construction of $g_{\varepsilon^{(i)}}$, $P^{n}_{g_{\varepsilon^{(i)}}}\ll P^{n}_{g_{0}}$. Then it follows from Lemma 2.5 (Fano’s lemma) that

$$\sup_{0\leq i\leq M} P^{n}_{g_{\varepsilon^{(i)}}} \biggl( \Vert \hat {f}_{n}-g_{\varepsilon^{(i)}} \Vert _{p} \geq \frac{\delta_{j}}{2} \biggr)\geq\sup_{0\leq i\leq M} P^{n}_{g_{\varepsilon^{(i)}}} \bigl(A^{c}_{\varepsilon^{(i)}}\bigr) \geq\min \biggl\{ \frac{1}{2}, \sqrt{M}e^{-\frac{3}{e}}e^{-\mathcal {K}_{M}} \biggr\} . $$

Furthermore,

$$E \Vert \hat{f}_{n}-g_{\varepsilon^{(i)}} \Vert _{p}^{p} \geq \frac{\delta_{j}^{p}}{2^{p}}P^{n}_{g_{\varepsilon^{(i)}}} \biggl( \Vert \hat {f}_{n}-g_{\varepsilon^{(i)}} \Vert _{p}\geq \frac{\delta_{j}}{2} \biggr)\geq2^{-2psj} P^{n}_{g_{\varepsilon ^{(i)}}} \bigl(A^{c}_{\varepsilon^{(i)}}\bigr). $$

Taking $2^{j}\sim n^{\frac{1}{2(2s+1)}}$, we obtain that

3.2 $$\begin{aligned} \sup_{0\leq i\leq M} E \Vert \hat{f}_{n}-g_{\varepsilon^{(i)}} \Vert _{p}^{p}\geq 2^{-2psj} \sup _{0\leq i\leq M}P^{n}_{g_{\varepsilon ^{(i)}}}\bigl(A^{c}_{\varepsilon^{(i)}} \bigr)\geq n^{-\frac{ps}{2s+1}} \min \biggl\{ \frac{1}{2}, \sqrt{M}e^{-\frac {3}{e}}e^{-\mathcal{K}_{M}} \biggr\} \end{aligned}$$

with $\mathcal{K}_{M}:=\inf_{0\leq v\leq M} \frac{1}{M}\sum_{i\neq v}K(P^{n}_{g_{\varepsilon^{(i)}}}, P^{n}_{g_{\varepsilon^{(v)}}})$. By the definition of Kullback divergence,

3.3 $$\begin{aligned} K\bigl(P^{n}_{g_{\varepsilon^{(i)}}},P^{n}_{g_{0}}\bigr)&= \int _{\mathbb{R}^{2n}} \biggl[\ln\frac{\prod_{i=1}^{n}g_{\varepsilon^{(i)}}(x_{i},y_{i})}{\prod_{i=1}^{n}g_{0} (x_{i},y_{i})} \biggr]\prod _{i=1}^{n}g_{\varepsilon^{(i)}}(x_{i},y_{i}) \,dx_{1}\,dy_{1}\,dx_{2}\,dy_{2}\cdots \,dx_{n}\,dy_{n} \\ &=n \int_{\mathbb{R}^{2}} g_{\varepsilon^{(i)}}(x_{1},y_{1}) \ln\frac {g_{\varepsilon ^{(i)}}(x_{1},y_{1})}{g_{0}(x_{1},y_{1})}\,dx_{1}\,dy_{1} \\ &\leq n \int_{\mathbb{R}^{2}} g_{\varepsilon^{(i)}}(x_{1},y_{1}) \biggl[\frac {g_{\varepsilon^{(i)}}(x_{1},y_{1})}{g_{0}(x_{1},y_{1})}-1 \biggr]\,dx_{1}\,dy_{1}, \end{aligned}$$

where we applied the inequality $\ln u\leq u-1$ for $u>0$ in the last inequality. Note that

$$\begin{aligned} &\int_{\mathbb{R}^{2}} g_{\varepsilon^{(i)}}(x_{1},y_{1}) \biggl[\frac {g_{\varepsilon^{(i)}}(x_{1},y_{1})}{g_{0}(x_{1},y_{1})}-1 \biggr]\,dx_{1}\,dy_{1}\\ &\quad = \int_{\mathbb{R}^{2}} \bigl[g_{0}(x_{1},y_{1}) \bigr]^{-1}\bigl[g_{\varepsilon ^{(i)}}(x_{1},y_{1})- g_{0}(x_{1},y_{1})\bigr]^{2}\,dx_{1}\,dy_{1} \end{aligned} $$

and $g_{0}(x_{1},y_{1})=c_{0}$ for $(x_{1},y_{1})\in[0,2N-1]\times [-N+1,N]$. Combining this with the Parseval identity, we reduce (3.3) to

3.4 $$\begin{aligned} K\bigl(P^{n}_{g_{\varepsilon^{(i)}}},P^{n}_{g_{0}}\bigr) \leq n c_{0}^{-1}a_{j}^{2} \biggl\Vert \sum_{k\in\Delta_{j}}\varepsilon_{k}^{i} \psi^{1}_{j,k}(x,y) \biggr\Vert _{2}^{2} = n c_{0}^{-1}a_{j}^{2}\sum _{k\in\Delta_{j}} \bigl\vert \varepsilon_{k}^{i} \bigr\vert ^{2} \leq n c_{0}^{-1}a_{j}^{2}2^{2j}. \end{aligned}$$

Hence

$$\mathcal{K}_{M}\leq \frac{1}{M}\sum _{i=1}^{M}K\bigl(P^{n}_{g_{\varepsilon^{(i)}}}, P^{n}_{g_{0}}\bigr)\leq c_{0}^{-1}na_{j}^{2}2^{2j}. $$

On the other hand, $2^{j}\sim n^{\frac{1}{2(2s+1)}}$ implies $na_{j}^{2}\leq C$. Then it follows from $M\geq2^{\frac {2^{2j}}{8}}\geq e^{{2^{2j}\frac{\ln2}{8}}}$ that

$$\sqrt{M}e^{-\mathcal{K}_{M}}\geq e^{2^{2j}\frac{\ln 2}{16}-c_{0}^{-1}C2^{2j}}\geq1 $$

by choosing $C>0$ such that $C<\frac{\ln2}{16}c_{0}$. This with (3.2) leads to

3.5 $$\begin{aligned} \sup_{0\leq i\leq M} E \Vert \hat{f}_{n}-g_{\varepsilon^{(i)}} \Vert _{p}^{p}\geq n^{-\frac{ps}{2s+1}} \min \biggl\{ \frac{1}{2}, \sqrt{M}e^{-\frac {3}{e}}e^{-\mathcal{K}_{M}} \biggr\} \gtrsim n^{-\frac{ps}{2s+1}}. \end{aligned}$$

Now, it remains to show that

3.6 $$\begin{aligned} \sup_{f_{\ast}\in B_{r,q}^{s}(H)} E \Vert \hat{f}_{n}-f_{\ast} \Vert _{p}^{p}\geq C \biggl(\frac{\ln n}{n} \biggr)^{\frac{(s-\frac{2}{r}+\frac {2}{p})p}{2(s-\frac{2}{r})+1}}. \end{aligned}$$

Similarly to the proof of (3.5), we construct the family of density functions $\{g_{k}, k\in\Delta_{j}\}$ as follows:

$$g_{k}(x,y):=g_{0}(x,y)+a_{j} \psi^{1}_{j,k}(x,y), \quad k\in\Delta_{j}, $$

where $a_{j}:=2^{-j(s+1-\frac{2}{r})}$. Obviously, $\int_{\mathbb{R}^{2}} g_{k}(x,y) \,dx \,dy=\int_{\mathbb{R}^{2}} g_{0}(x,y) \,dx \,dy=1$, and

$$g_{k}(x, y) {|}_{[0,2N-1]\times[-N+1,N]}\geq c_{0}-2^{-j(s-\frac {2}{r})} \bigl\Vert \psi^{1} \bigr\Vert _{\infty}>0 $$

for large *j* since $s>\frac{2}{r}$. Then $g_{k}$ is a bivariate density function for fixed $k\in\Delta_{j}$. From the proof of (3.5) we know that $g_{0}\in B^{s}_{r,q}(H)$. This with

$$\bigl\Vert a_{j}\psi^{1}_{j,k} \bigr\Vert _{B^{s}_{r,q}}\sim a_{j}2^{j(s+1-\frac {2}{r})}\leq1 $$

implies $g_{k}\in B^{s}_{r,q}(H)$ for $k\in\Delta_{j}$.

To prove (3.6), we need to show that

3.7 $$\begin{aligned} \sup_{k\in\Delta_{j}} E \Vert \hat{f}_{n}-g_{k} \Vert _{p}^{p}\geq C \biggl(\frac{\ln n}{n} \biggr)^{\frac{(s-\frac{2}{r}+\frac {2}{p})p}{2(s-\frac{2}{r})+1}}. \end{aligned}$$

When $k\neq k'\in\Delta_{j}$, $\operatorname{supp} \psi^{1}_{j,k} \cap \operatorname{supp} \psi ^{1}_{j,k'} =\emptyset$ and

$$\Vert g_{k}-g_{k'} \Vert _{p}^{p}=a_{j}^{p} \bigl\Vert \psi^{1}_{j,k}-\psi^{1}_{j,k'} \bigr\Vert _{p}^{p} =2a_{j}^{p}2^{j(p-2)} \bigl\Vert \psi^{1} \bigr\Vert _{p}^{p}=2 \cdot2^{-j(s-\frac {2}{r}+\frac{2}{p})p} \bigl\Vert \psi^{1} \bigr\Vert _{p}^{p}. $$

Moreover,

$$\Vert g_{k}-g_{k'} \Vert _{p}=2^{\frac{1}{p}} \bigl\Vert \psi^{1} \bigr\Vert _{p} 2^{-j(s-\frac {2}{r}+\frac{2}{p})}:= \delta_{j}. $$

Define $B_{k}:= \{\|\hat{f}_{n}-g_{k}\|_{p}<\frac{\delta _{j}}{2} \}$. Then $B_{k}\cap B_{k'}=\emptyset$ ($k\neq k'$). According to Lemma 2.5 (Fano’s lemma), we find that

3.8 $$\begin{aligned} \sup_{k\in\Delta_{j}}P_{g_{k}}^{n} \biggl( \Vert \hat {f}_{n}-g_{k} \Vert _{p}\geq \frac{\delta_{j}}{2} \biggr) \geq\min \biggl\{ \frac{1}{2}, \sqrt{M}e^{-3e^{-1}}e^{-\mathcal {K}_{M}} \biggr\} , \end{aligned}$$

where $M=\sharp\Delta_{j}$ and $\mathcal{K}_{M}:=\inf_{0\leq v\leq M}\frac{1}{M}\sum_{k\neq v} K(P^{n}_{g_{k}}, P^{n}_{g_{v}})\leq\frac{1}{M}\sum_{k\neq 0}K(P^{n}_{g_{k}}, P^{n}_{g_{0}})$. Similar to (3.3)–(3.4), we conclude that

$$K\bigl(P^{n}_{g_{k}}, P^{n}_{g_{v}}\bigr) \leq n \int_{\mathbb{R}^{2}} \bigl[g_{0}(x,y)\bigr]^{-1} \bigl[g_{k}(x,y)-g_{0}(x,y)\bigr]^{2} \,dx \,dy\leq c_{0}^{-1}C_{1}na_{j}^{2}. $$

Hence $\mathcal{K}_{M}\leq c^{-1}_{0}C_{1}na_{j}^{2}$. By taking $2^{j}\sim(\frac{n}{\ln n})^{\frac{1}{2(s-\frac {2}{r})+1}}$we obtain that $\ln2^{j}\geq C'\ln n$ and $e^{-\mathcal{K}_{M}}\geq e^{-c^{-1}_{0}C_{1}na_{j}^{2}}\geq e^{-c_{0}^{-1}C\ln n}$, thanks to $na_{j}^{2}\leq C_{2}\ln n$ ($C=C_{1}C_{2}$). Moreover, choosing $C_{1}$ and $C'$ such that $C'>c_{0}^{-1}C$, we have

$$\sqrt{M}e^{-3e^{-1}}e^{-\mathcal{K}_{M}} \gtrsim e^{\ln2^{j}}e^{-3e^{-1}}e^{-\mathcal{K}_{M}} \geq e^{C'\ln n-c_{0}^{-1}C\ln n-3e^{-1}}\gtrsim1 $$

due to $M\sim2^{2j}$. This with (3.8) implies $\sup_{k\in\Delta_{j}}P_{g_{k}}^{n}(\|\hat{f}_{n}-g_{k}\|_{p}\geq\frac {\delta_{j}}{2}) \gtrsim1$. Furthermore,

$$\sup_{k\in\Delta_{j}} E \Vert \hat{f}_{n}-g_{k} \Vert _{p}^{p} \geq\frac{\delta_{j}^{p}}{2^{p}}P_{g_{k}} \biggl( \Vert \hat{f}_{n}-g_{k} \Vert _{p} \geq\frac{\delta_{j}}{2} \biggr) e^{-3e^{-1}}\gtrsim\delta_{j}^{p}. $$

Then the desired conclusion (3.7) follows from $\delta_{j}:=2^{\frac{1}{p}}\|\psi^{1}\|_{p}2^{-j(s-\frac{2}{r}+\frac {2}{p})}$ and the choice of $2^{j}\sim(\frac{n}{\ln n})^{\frac{1}{2(s-\frac {2}{r})+1}}$. This completes the proof. □

## Proofs of upper bounds

In this section, we prove the upper bounds of wavelet estimators. The result of the linear one is derived firstly. We restate and prove Theorem 1.2 as Theorem 4.1.

### Theorem 4.1

*Let* $\hat{f}^{{\mathrm{lin}}}_{n}$ *be the linear estimator of* $f_{\ast}\in B_{r,q}^{s}(H, Q)$ *defined in* (1.3) *with* $1\leq r,q<\infty$, $s>0$. *If the density of* *X* *is bounded*, *then for* $\{r\geq p\geq1\}$ *or* $\{r\leq p<\infty\textit{ and } s>\frac{2}{r}\}$,

$$\sup_{f_{\ast}\in B_{r,q}^{s}(H, Q)} E \bigl\Vert \hat{f}^{{\mathrm{lin}}}_{n}-f_{\ast} \bigr\Vert _{p}^{p}\lesssim n^{-\frac {ps'}{2s'+1}} $$

*with* $s'=s-(\frac{2}{r}-\frac{2}{p})_{+}$ *and* $x_{+}:=\max\{x,0\}$.

### Proof

When $r\leq p$, $s':=s-(\frac{2}{r}-\frac{2}{p})_{+}=s-\frac {2}{r}+\frac{2}{p}$ and $B_{r,q}^{s}(\mathbb{R}^{2})\hookrightarrow B_{p,q}^{s'}(\mathbb{R}^{2})$ thanks to Remark 1.2. Then

$$\sup_{f_{\ast}\in B_{r,q}^{s}(H, Q)} E \bigl\Vert \hat{f}^{{\mathrm{lin}}}_{n}-f_{\ast} \bigr\Vert _{p}^{p}\lesssim\sup_{f_{\ast }\in B_{r,q}^{s'}(H, Q)} E \bigl\Vert \hat{f}^{{\mathrm{lin}}}_{n}-f_{\ast} \bigr\Vert _{p}^{p}. $$

When $r>p$ and $f_{\ast}$ has a compact support, then $\hat {f}_{n}^{{\mathrm{lin}}}$ does due to *φ* having the same property. By the Hölder inequality,

$$\sup_{f_{\ast}\in B_{r,q}^{s}(H, Q)} E \bigl\Vert \hat{f}^{{\mathrm{lin}}}_{n}-f_{\ast} \bigr\Vert _{p}^{p}\lesssim\sup_{f_{\ast }\in B_{r,q}^{s}(H, Q)} \bigl(E \bigl\Vert \hat{f}^{{\mathrm{lin}}}_{n}-f_{\ast} \bigr\Vert _{r}^{r}\bigr)^{\frac{p}{r}}. $$

Because $s'=s$ in that case, it is sufficient to prove that

4.1 $$\begin{aligned} \sup_{f_{\ast}\in B_{r,q}^{s'}(H, Q)} E \bigl\Vert \hat{f}^{{\mathrm{lin}}}_{n}-f_{\ast} \bigr\Vert _{p}^{p}\lesssim n^{-\frac {ps'}{2s'+1}} \end{aligned}$$

for the conclusion of Theorem 4.1.

Recall that $\hat{f}^{{\mathrm{lin}}}_{n}:=\sum_{k\in\wedge _{j_{0}}}\hat{\alpha}_{j_{0},k}\phi_{j_{0},k}$. Then by Lemma 2.1 we conclude that

$$E \bigl\Vert \hat{f}_{n}^{{\mathrm{lin}}}-E\bigl( \hat{f}_{n}^{{\mathrm{lin}}}\bigr) \bigr\Vert _{p}^{p}=E \biggl\Vert \sum_{k\in\wedge_{j_{0}}}(\hat{\alpha}_{j_{0},k}- \alpha_{j_{0},k})\varphi _{j_{0},k} \biggr\Vert _{p}^{p} \lesssim2^{j_{0}(p-2)}\sum_{k\in\wedge_{j_{0}}} E \vert \hat{ \alpha }_{j_{0},k}-\alpha_{j_{0},k} \vert ^{p} $$

due to Lemma 1.2. It follows from Lemma 2.2 and $\sharp\wedge_{j_{0}}\lesssim 2^{2j_{0}}$ that

4.2 $$\begin{aligned} E \bigl\Vert \hat{f}_{n}^{{\mathrm{lin}}}-E\bigl( \hat{f}_{n}^{{\mathrm {lin}}}\bigr) \bigr\Vert _{p}^{p} \lesssim2^{j_{0}(p-2)} 2^{2j_{0}} 2^{-\frac {pj_{0}}{2}}n^{-\frac{p}{2}} \lesssim2^{\frac{pj_{0}}{2}}n^{-\frac {p}{2}}\lesssim n^{-\frac{ps'}{2s'+1}} \end{aligned}$$

thanks to the choice of $2^{j_{0}}\sim n^{\frac{1}{2s'+1}}$.

On the other hand, by Lemma 2.1, $E(\hat{f}^{{\mathrm{lin}}}_{n})=\sum_{k\in\wedge_{j_{0}}}\alpha _{j_{0},k}\varphi_{j_{0},k}=P_{j_{0}}f_{\ast}$. Combining this with $f_{\ast}\in B_{p,q}^{s'}(\mathbb{R}^{2})$ and Remark 1.1, we get that

$$\bigl\Vert E\bigl(\hat{f}^{{\mathrm{lin}}}_{n}\bigr)-f_{\ast}\bigr\Vert _{p}^{p}= \Vert P_{j_{0}}f_{\ast}-f_{\ast} \Vert _{p}^{p}\lesssim2^{-j_{0}ps'}. $$

Taking $2^{j_{0}}\sim n^{\frac{1}{2s'+1}}$, it is easy to show

4.3 $$\begin{aligned} \bigl\Vert E\bigl(\hat{f}^{{\mathrm{lin}}}_{n}\bigr)-f_{\ast}\bigr\Vert _{p}^{p}\lesssim n^{-\frac{ps'}{2s'+1}}. \end{aligned}$$

Hence, by (4.2)–(4.3),

$$\begin{aligned} \sup_{f_{\ast}\in B_{r,q}^{s'}(H, Q)} E \bigl\Vert \hat{f}^{{\mathrm{lin}}}_{n}-f_{\ast} \bigr\Vert _{p}^{p} &\lesssim\sup_{f_{\ast}\in B_{r,q}^{s'}(H, Q)}E \bigl\Vert \hat{f}_{n}^{{\mathrm {lin}}}-E\bigl(\hat{f}_{n}^{{\mathrm{lin}}} \bigr) \bigr\Vert _{p}^{p}+\sup_{f_{\ast}\in B_{r,q}^{s'}(H, Q)} \bigl\Vert E\bigl(\hat{f}^{{\mathrm{lin}}}_{n}\bigr)-f_{\ast}\bigr\Vert _{p}^{p} \\ &\lesssim n^{-\frac{ps'}{2s'+1}}, \end{aligned}$$

which means that (4.1) holds. The proof is done. □

Next, we are in a position to prove the conclusion of the nonlinear one.

### Theorem 4.2

*Let* $\hat{f}^{{\mathrm{non}}}_{n}$ *be the nonlinear estimator of* $f_{\ast}\in B_{r,q}^{s}(H, Q)$ *defined in* (1.4) *with* $1\leq r,q<\infty$, $s>0$. *If the density of* *X* *is bounded*, *then for* $\{r\geq p\geq1\}$ *or* $\{r\leq p<\infty\textit{ and } s>\frac{2}{r}\}$,

$$\sup_{f_{\ast}\in B_{r,q}^{s}(H, Q)} E \bigl\Vert \hat{f}^{{\mathrm {non}}}_{n}-f_{\ast} \bigr\Vert _{p}^{p}\lesssim (\ln n)^{p} \biggl( \frac{\ln n}{n} \biggr)^{\alpha p} $$

*with* $\alpha:=\min \{\frac{s}{2s+1}, \frac{s-\frac{2}{r}+\frac{2}{p}}{2(s-\frac {2}{r})+1} \}$.

### Proof

We only need to prove the case $r\leq p$. In fact, when $r>p$, $\hat{f}^{{\mathrm{non}}}_{n}$ has a compact support because of *φ*, *ψ*, and $f_{\ast}$ have the same property. By the Hölder inequality,

$$\sup_{f_{\ast}\in B_{r,q}^{s}(H, Q)}E \bigl\Vert \hat{f}^{{\mathrm {non}}}_{n}-f_{\ast} \bigr\Vert _{p}^{p}\lesssim \sup_{f_{\ast}\in B_{r,q}^{s}(H, Q)} \bigl(E \bigl\Vert \hat{f}^{{\mathrm {non}}}_{n}-f_{\ast} \bigr\Vert _{r}^{r}\bigr)^{\frac{p}{r}}. $$

Using Theorem 4.2 for the case $r=p$, we find that $\sup_{f_{\ast}\in B_{r,q}^{s}(H,Q)} E\|\hat{f}^{{\mathrm {non}}}_{n}-f_{\ast}\|_{r}^{r}\lesssim [4] (\ln n)^{r}(\frac{\ln n}{n})^{\alpha r}$, and therefore

$$\sup_{f_{\ast}\in B_{r,q}^{s}(H,Q)} E \bigl\Vert \hat{f}^{{\mathrm{non}}}_{n}-f_{\ast} \bigr\Vert _{p}^{p}\lesssim(\ln n)^{p} \biggl( \frac{\ln n}{n} \biggr)^{\alpha p}. $$

It remains to estimate the case $r\leq p$. Recall that

$$\hat{f}_{n}^{{\mathrm{non}}}-f_{\ast}=\bigl(\hat {f}_{n}^{lin}-P_{j_{0}}f_{\ast} \bigr)+(P_{j_{1}+1}f_{\ast}-f_{\ast}) +\sum _{j=j_{0}}^{j_{1}}\sum_{i=1}^{3} \sum_{k\in\wedge_{j}} \bigl(\hat{\beta}^{i}_{j,k}1_{\{|\hat{\beta}^{i}_{j,k}|>\lambda_{j}\} }- \beta^{i}_{j,k} \bigr)\psi^{i}_{j,k} $$

with $\lambda_{j}=T2^{-\frac{j}{2}}\sqrt{\frac{\ln n}{n}}$. Denote $f_{j_{0},j_{1}}:=\sum_{j=j_{0}}^{j_{1}}\sum_{i=1}^{3}\sum_{k\in\wedge_{j}} (\hat{\beta}^{i}_{j,k}1_{\{|\hat{\beta}^{i}_{j,k}|>\lambda_{j}\}}-\beta ^{i}_{j,k})\psi^{i}_{j,k}$. Then

4.4 $$\begin{aligned} E \bigl\Vert \hat{f}_{n}^{{\mathrm{non}}}-f_{\ast} \bigr\Vert _{p}^{p}\lesssim E \bigl\Vert \hat{f}_{n}^{{\mathrm{lin}}}-P_{j_{0}}f_{\ast} \bigr\Vert _{p}^{p}+ \Vert P_{j_{1}+1}f_{\ast}-f_{\ast} \Vert _{p}^{p} +E \Vert f_{j_{0},j_{1}} \Vert _{p}^{p}. \end{aligned}$$

From the proof of Theorem 4.1 we obtain that

$$E \bigl\Vert \hat{f}_{n}^{{\mathrm{lin}}}-P_{j_{0}}f_{\ast} \bigr\Vert _{p}^{p} \lesssim2^{\frac{j_{0}p}{2}}n^{-\frac{p}{2}} \lesssim \biggl(\frac{\ln n}{n} \biggr)^{\alpha p} $$

and

4.5 $$\begin{aligned} \Vert P_{j_{1}+1}f_{\ast}-f_{\ast} \Vert _{p}^{p}\lesssim 2^{-j_{1}ps'}\lesssim \biggl( \frac{\ln n}{n} \biggr)^{\alpha p} \end{aligned}$$

due to $2^{j_{0}}\sim n^{\frac{1}{2m+1}}$, $2^{j_{1}}\sim\frac{n}{\ln n}$ and $\alpha=\min \{\frac{s}{2s+1}, \frac{s-\frac{2}{r}+\frac{2}{p}}{2(s-\frac{2}{r})+1} \}$.

By $f_{j_{0},j_{1}}:= \sum_{j=j_{0}}^{j_{1}}\sum_{i=1}^{3}\sum_{k\in\wedge_{j}} (\hat{\beta}^{i}_{j,k}1_{\{|\hat{\beta}^{i}_{j,k}|>\lambda_{j}\}}-\beta ^{i}_{j,k})\psi^{i}_{j,k}$ and Lemma 1.2,

$$E \Vert f_{j_{0},j_{1}} \Vert _{p}^{p}\lesssim (j_{1}-j_{0}+1)^{p-1}\sum _{j=j_{0}}^{j_{1}}\sum_{i=1}^{3}2^{j(p-2)} \sum_{k\in\wedge_{j}}E \bigl\vert \hat{\beta}^{i}_{j,k}1_{\{ \vert \hat{\beta }^{i}_{j,k} \vert >\lambda_{j}\}} -\beta^{i}_{j,k} \bigr\vert ^{p}. $$

On the other hand, it is easy to see that

$$\begin{aligned} \bigl\vert \hat{\beta}^{i}_{j,k}1_{\{ \vert \hat{\beta }^{i}_{j,k} \vert >\lambda_{j}\}}- \beta^{i}_{j,k} \bigr\vert &= \bigl\vert \hat{\beta }^{i}_{j,k}-\beta^{i}_{j,k} \bigr\vert (1_{\{ \vert \hat{\beta}^{i}_{j,k} \vert \geq\lambda_{j}, \vert \beta ^{i}_{j,k} \vert < \lambda_{j}/2\}} +1_{\{ \vert \hat{\beta}^{i}_{j,k} \vert \geq\lambda_{j}, \vert \beta^{i}_{j,k} \vert \geq \lambda_{j}/2\}} ) \\ &\quad {}+ \bigl\vert \beta^{i}_{j,k} \bigr\vert (1_{\{ \vert \hat{\beta}^{i}_{j,k} \vert < \lambda_{j}, \vert \beta ^{i}_{j,k} \vert >2\lambda_{j}\}} +1_{\{ \vert \hat{\beta}^{i}_{j,k} \vert < \lambda_{j}, \vert \beta^{i}_{j,k} \vert \leq 2\lambda_{j}\}} ) \end{aligned}$$

and $1_{\{|\hat{\beta}^{i}_{j,k}|\geq\lambda_{j}, |\beta ^{i}_{j,k}|<\lambda_{j}/2\}}\leq1_{\{|\hat{\beta}^{i}_{j,k}-\beta ^{i}_{j,k}|>\lambda_{j}/2\}}$. Then

4.6 $$\begin{aligned} E \Vert f_{j_{0},j_{1}} \Vert _{p}^{p}\lesssim T_{1}+T_{2}+T_{3}+T_{4} \end{aligned}$$

with

$$\begin{aligned}& T_{1}:=(\ln n)^{p-1}\sum_{j=j_{0}}^{j_{1}} \sum_{i=1}^{3}2^{j(p-2)} \sum _{k\in\wedge_{j}}E \bigl[ \bigl\vert \hat{ \beta}^{i}_{j,k}-\beta ^{i}_{j,k} \bigr\vert ^{p} 1_{\{ \vert \hat{\beta}^{i}_{j,k}-\beta^{i}_{j,k} \vert >\lambda_{j}/2\}} \bigr], \\& T_{2}:=(\ln n)^{p-1}\sum_{j=j_{0}}^{j_{1}} \sum_{i=1}^{3}2^{j(p-2)} \sum _{k\in\wedge_{j}}E \bigl[ \bigl\vert \hat{ \beta}^{i}_{j,k}-\beta ^{i}_{j,k} \bigr\vert ^{p} 1_{\{ \vert \hat{\beta}^{i}_{j,k} \vert \geq\lambda_{j}, \vert \beta^{i}_{j,k} \vert \geq \lambda_{j}/2\}} \bigr], \\& T_{3}:=(\ln n)^{p-1}\sum_{j=j_{0}}^{j_{1}} \sum_{i=1}^{3}2^{j(p-2)} \sum _{k\in\wedge_{j}}E \bigl[ \bigl\vert \beta^{i}_{j,k} \bigr\vert ^{p} 1_{\{ \vert \hat{\beta}^{i}_{j,k} \vert < \lambda_{j}, \vert \beta^{i}_{j,k} \vert \leq2\lambda _{j}\}} \bigr], \\& T_{4}:=(\ln n)^{p-1}\sum_{j=j_{0}}^{j_{1}} \sum_{i=1}^{3}2^{j(p-2)} \sum _{k\in\wedge_{j}}E \bigl[ \bigl\vert \beta^{i}_{j,k} \bigr\vert ^{p} 1_{\{ \vert \hat{\beta}^{i}_{j,k} \vert < \lambda_{j}, \vert \beta^{i}_{j,k} \vert > 2\lambda _{j}\}} \bigr]. \end{aligned}$$

When $|\hat{\beta}^{i}_{j,k}|<\lambda_{j}$ and $|\beta^{i}_{j,k}|>2\lambda_{j}$, $|\hat{\beta}^{i}_{j,k}-\beta^{i}_{j,k} |\geq |\beta^{i}_{j,k}|-|\hat{\beta}^{i}_{j,k}|>{|\hat{\beta }^{i}_{j,k}|}/{2}$. Hence

$$\bigl\vert \beta^{i}_{j,k} \bigr\vert ^{p}1_{\{ \vert \hat{\beta}^{i}_{j,k} \vert < \lambda_{j}, \vert \beta^{i}_{j,k} \vert > 2\lambda_{j}\}}\lesssim \bigl\vert \hat{\beta}^{i}_{j,k} -\beta^{i}_{j,k} \bigr\vert ^{p}1_{\{ \vert \hat{\beta}^{i}_{j,k}-\beta ^{i}_{j,k} \vert >\lambda_{j}/2\}}. $$

Then (4.6) reduces to

4.7 $$\begin{aligned} E \Vert f_{j_{0},j_{1}} \Vert _{p}^{p}\lesssim T_{1}+T_{2}+T_{3}. \end{aligned}$$

By (4.4)–(4.5) and (4.7) it is sufficient to show

4.8 $$\begin{aligned} T_{\ell}\lesssim(\ln n)^{p} \biggl(\frac{\ln n}{n} \biggr)^{\alpha p},\quad \ell=1, 2, 3, \end{aligned}$$

for the conclusion of Theorem 4.2.

To estimate $T_{1}$, using the Hölder inequality, we find that

$$T_{1}\lesssim (\ln n)^{p-1}\sum _{j=j_{0}}^{j_{1}}\sum_{i=1}^{3}2^{j(p-2)} \sum_{k\in \wedge_{j}} \bigl( E \bigl\vert \hat{ \beta}^{i}_{j, k}-\beta^{i}_{j, k} \bigr\vert ^{2p} \bigr)^{\frac{1}{2}} \bigl[E ( 1_{\{ \vert \hat{\beta}^{i}_{j, k}-\beta^{i}_{j, k} \vert \geq \lambda_{j}/2\}} ) \bigr]^{\frac{1}{2}}. $$

Note that $E (1_{\{|\hat{\beta}^{i}_{j, k}-\beta^{i}_{j, k}|\geq \lambda_{j}/2\}} )= P (|\hat{\beta}^{i}_{j,k}-\beta^{i}_{j, k}|\geq\frac{\lambda _{j}}{2} )\leq 2^{-\varepsilon j}$ due to Lemma 2.3. Taking *ε* such that $\varepsilon>p$, we conclude that

$$T_{1}\lesssim (\ln n)^{p-1} n^{-\frac{p}{2}}\sum _{j=j_{0}}^{j_{1}}\sum_{i=1}^{3}2^{\frac {p-\varepsilon}{2}j} \lesssim(\ln n)^{p-1}n^{-\frac{p}{2}} 2^{\frac{p}{2}j_{0}}\lesssim(\ln n)^{p-1}n^{-\frac{ps}{2s+1}} $$

thanks to Lemma 2.2, $\sharp\wedge_{j}\lesssim2^{2j}$ and the choice of $j_{0}$. Hence (4.8) with $\ell=1$ holds since $\alpha\leq\frac {s}{2s+1}$.

To estimate $T_{2}$ and $T_{3}$, define

$$2^{j_{0}^{\ast}}\sim \biggl(\frac{n}{\ln n} \biggr)^{1-2\alpha }, \qquad 2^{j_{1}^{\ast}} \sim \biggl(\frac{n}{\ln n} \biggr)^{\frac{\alpha}{s-\frac{2}{r}+\frac{2}{p}}}. $$

Recall that $2^{j_{0}}\sim n^{\frac{1}{2m+1}}$, $2^{j_{1}}\sim\frac {n}{\ln n}$ and $\alpha:=\min \{ \frac{s}{2s+1}, \frac{s-\frac{2}{r}+\frac {2}{p}}{2(s-\frac{2}{r})+1} \}$. Then

$$1-2\alpha\geq\frac{1}{2s+1}>\frac{1}{2m+1} \quad \text{and}\quad \frac{\alpha }{s-\frac{2}{r}+ \frac{2}{p}}\leq\frac{1}{2(s-\frac{2}{r})+1}\leq1. $$

Hence $2^{j_{0}}\leq2^{j_{0}^{\ast}}$ and $2^{j_{1}^{\ast}}\leq2^{j_{1}}$. Moreover, a simple computation shows that $1-2\alpha\leq\frac{\alpha}{s-\frac{2}{r}+\frac{2}{p}}$, which implies $2^{j_{0}^{\ast}}\leq2^{j_{1}^{\ast}}$.

Now, we estimate $T_{2}$ by dividing $T_{2}$ into

4.9 $$\begin{aligned} T_{2}&=(\ln n)^{p-1} \Biggl(\sum _{j=j_{0}}^{j_{0}^{\ast }}+\sum_{j=j_{0}^{\ast}+1}^{j_{1}} \Biggr)\sum_{i=1}^{3}2^{j(p-2)} \sum _{k\in\wedge_{j}}E \bigl[ \bigl\vert \hat{ \beta}^{i}_{j,k}-\beta ^{i}_{j,k} \bigr\vert ^{p} 1_{\{ \vert \hat{\beta}^{i}_{j,k} \vert \geq\lambda_{j}, \vert \beta^{i}_{j,k} \vert \geq\lambda _{j}/2\}} \bigr] \\ &:=t_{1}+t_{2}. \end{aligned}$$

Since $1_{\{|\hat{\beta}^{i}_{j, k}|\geq\lambda_{j},|\beta^{i}_{j, k}|\geq\lambda_{j}/2 \}}\leq1$, by Lemma 2.2 we know that

4.10 $$\begin{aligned} t_{1}\lesssim(\ln n)^{p-1} \sum_{j=j_{0}}^{j_{0}^{\ast}} \sum_{i=1}^{3} 2^{\frac{ p j}{2}}n^{-\frac{p}{2}} \lesssim (\ln n)^{p-1}n^{-\frac{p}{2}}2^{\frac{j_{0}^{\ast}}{2}p}\lesssim (\ln n)^{p} \biggl(\frac{\ln n}{n} \biggr)^{\alpha p} \end{aligned}$$

thanks to $\sharp\wedge_{j}\lesssim2^{2j}$ and the choice of $j_{0}^{*}$. To estimate $t_{2}$, we observe that

$$1_{ \{ \vert \hat{\beta}^{i}_{j, k} \vert \geq\lambda_{j}, \vert \beta^{i}_{j, k} \vert \geq \frac{\lambda_{j}}{2} \}}\leq1_{ \{ \vert \beta^{i}_{j, k} \vert \geq\frac {\lambda_{j}}{2} \}} \lesssim \biggl(\frac{ \vert \beta^{i}_{j, k} \vert }{\lambda_{j}} \biggr)^{r}. $$

This with Lemma 2.2 leads to

4.11 $$\begin{aligned} t_{2}\lesssim(\ln n)^{p-1} \sum_{j=j_{0}^{\ast}+1}^{j_{1}} \sum_{i=1}^{3}2^{j(\frac {p}{2}-2)}n^{-\frac{p}{2}} \sum_{k\in\wedge_{j}} \biggl(\frac{ \vert \beta^{i}_{j, k} \vert }{\lambda_{j}} \biggr)^{r}. \end{aligned}$$

Note that $\|\beta_{j, \cdot}\|_{r}\lesssim2^{-j(s+1-\frac{2}{r})}$ because of $f_{\ast}\in B^{s}_{r,q}$ and Lemma 1.1. Then (4.11) reduces to

4.12 $$\begin{aligned} t_{2}\lesssim(\ln n)^{p-\frac{r}{2}-1}n^{\frac{r-p}{2}}\sum _{j=j_{0}^{\ast}+1}^{j_{1}} 2^{-j(sr+\frac{r}{2}-\frac{p}{2})} \end{aligned}$$

thanks to $\lambda_{j}=\frac{T}{2}2^{-\frac{j}{2}}\sqrt{\frac{\ln n}{n}}$. Denote $\theta:=sr+\frac{r}{2}-\frac{p}{2}$. When $\theta>0$, $r>\frac{p}{2s+1}$ and

4.13 $$\begin{aligned} t_{2}\lesssim (\ln n)^{p-\frac{r}{2}-1}n^{\frac{r-p}{2}} 2^{-j_{0}^{\ast}(sr+\frac{r}{2}-\frac{p}{2})}\lesssim (\ln n)^{p} \biggl(\frac{\ln n}{n} \biggr)^{\alpha p} \end{aligned}$$

due to the choice of $j_{0}^{\ast}$. In (4.13), we use the fact $\alpha=\frac{s}{2s+1}$ in the case $r>\frac{p}{2s+1}$.

To show (4.13) for $\theta\leq0$, define $r_{1}:=(1-2\alpha)p>0$. Then $\alpha=\frac{s-\frac{2}{r}+\frac {2}{p}}{2(s-\frac{2}{r})+1}\leq\frac{s}{2s+1}$ and $r\leq\frac{p}{2s+1}\leq(1-2\alpha)p=r_{1}$ because $\theta\leq0$. The same arguments as (4.11) show that

$$t_{2}\lesssim(\ln n)^{p-1} \sum _{j=j_{0}^{\ast}+1}^{j_{1}}\sum_{i=1}^{3}2^{j(\frac {p}{2}-2)}n^{-\frac{p}{2}} \sum_{k\in\wedge_{j}} \biggl(\frac{ \vert \beta^{i}_{j, k} \vert }{\lambda_{j}} \biggr)^{r_{1}}. $$

It follows from $f_{\ast}\in B^{s}_{r, q}$ and Lemma 1.1 that

$$\Vert \beta_{j, \cdot} \Vert _{r_{1}}\leq \Vert \beta_{j, \cdot} \Vert _{r}\leq 2^{-j(s+1-\frac{2}{r})} $$

due to $r\leq r_{1}$. Therefore, similarly to (4.12), we get that

$$t_{2}\lesssim(\ln n)^{p-\frac{r_{1}}{2}-1}n^{\frac{r_{1}-p}{2}} \sum _{j=j_{0}^{\ast}+1}^{j_{1}} 2^{j [\frac{p-2}{2}-(s-\frac{2}{r}-\frac{1}{2})r_{1} ]}. $$

Note that $\frac{p}{2}-2-(s-\frac{2}{r}+\frac{1}{2})r_{1}=0$ because of $r_{1}=(1-2\alpha)p$ and $\alpha=\frac{s-\frac{2}{r}+\frac {2}{p}}{2(s-\frac{2}{r})+1}$. Then

4.14 $$\begin{aligned} t_{2}\lesssim(\ln n)^{p-\frac{r_{1}}{2}-1}n^{\frac {r_{1}-p}{2}}\lesssim (\ln n)^{p} \biggl(\frac{\ln n}{n} \biggr)^{\alpha p}, \end{aligned}$$

which implies that (4.13) holds for $\theta\leq0$. The desired conclusion (4.8) with $\ell=2$ follows from (4.9)–(4.10) and (4.13)–(4.14).

Finally, by splitting $T_{3}$ into

4.15 $$\begin{aligned} T_{3}&=(\ln n)^{p-1} \Biggl(\sum _{j=j_{0}}^{j_{0}^{\ast}}+\sum_{j=j_{0}^{\ast}+1}^{j_{1}} \Biggr)\sum_{i=1}^{3}2^{j(p-2)} \sum _{k\in\wedge_{j}}E \bigl[ \bigl\vert \beta^{i}_{j,k} \bigr\vert ^{p} 1_{\{ \vert \hat{\beta}^{i}_{j,k} \vert < \lambda_{j}, \vert \beta^{i}_{j,k} \vert \leq2\lambda _{j}\}} \bigr] \\ &:=e_{1}+e_{2} \end{aligned}$$

we obtain that

4.16 $$\begin{aligned} e_{1}\lesssim(\ln n)^{p-1}\sum_{j=j_{0}}^{j_{0}^{\ast }} \sum_{i=1}^{3} 2^{jp} \vert \lambda_{j} \vert ^{p} \lesssim(\ln n)^{\frac{3}{2}p-1}n^{-\frac{p}{2}}2^{\frac{j_{0}^{\ast }p}{2}} \lesssim (\ln n)^{p} \biggl(\frac{\ln n}{n} \biggr)^{\alpha p} \end{aligned}$$

thanks to $\sharp\wedge_{j}\lesssim2^{2j}$ and the choice of $\lambda_{j}$ and $j_{0}^{\ast}$.

To estimate $e_{2}$, we use the fact $1_{ \{|\hat{\beta}^{i}_{j, k}| \leq\lambda_{j}, |\beta^{i}_{j, k}|\leq2\lambda_{j} \}}\leq (\frac{2\lambda_{j}}{|\beta^{i}_{j, k}|} )^{p-r}$ because of $r\leq p$. Similarly to (4.11)–(4.13),

4.17 $$\begin{aligned} e_{2}\lesssim(\ln n)^{p} \biggl(\frac{\ln n}{n} \biggr)^{\alpha p} \end{aligned}$$

for $\theta>0$, where $\theta:=sr+\frac{r}{2}-\frac{p}{2}$. When $\theta\leq0$, we rewrite $e_{2}$ as follows:

4.18 $$\begin{aligned} e_{2}&=(\ln n)^{p-1} \Biggl(\sum _{j=j_{0}^{\ast}+1}^{j_{1}^{\ast}}+\sum_{j=j_{1}^{\ast}+1}^{j_{1}} \Biggr)\sum_{i=1}^{3}2^{j(p-2)} \sum _{k\in\wedge_{j}}E \bigl[ \bigl\vert \beta^{i}_{j,k} \bigr\vert ^{p} 1_{\{ \vert \hat{\beta}^{i}_{j,k} \vert < \lambda_{j}, \vert \beta^{i}_{j,k} \vert \leq2\lambda _{j}\}} \bigr] \\ &:=e_{1}^{\ast}+e_{2}^{\ast}. \end{aligned}$$

Proceeding as in (4.11) and (4.12), we find that

$$e_{1}^{\ast}\lesssim(\ln n)^{p-1} \biggl( \frac{\ln n}{n} \biggr)^{\frac{p-r}{2}} \sum_{j=j_{0}^{\ast}+1}^{j_{1}^{\ast}}2^{-j(sr+\frac{r-p}{2})} \lesssim(\ln n)^{p-1} \biggl(\frac{\ln n}{n} \biggr)^{\frac {p-r}{2}}2^{-j_{1}^{\ast} (sr+\frac{r-p}{2})}. $$

This with the choice of $2^{j_{1}^{\ast}}\sim(\frac{n}{\ln n})^{\frac {\alpha}{s-\frac{2}{r}+\frac{2}{p}}}$ leads to

4.19 $$\begin{aligned} e_{1}^{\ast}\lesssim(\ln n)^{p} \biggl( \frac{\ln n}{n} \biggr)^{\alpha p} \end{aligned}$$

due to $\alpha=\frac{s-\frac{2}{r}+\frac{2}{p}}{2(s-\frac{2}{r})+1}$ for $\theta\leq0$. When $r\leq p$,

$$\Vert \beta_{j, \cdot} \Vert _{p}\leq \Vert \beta_{j, \cdot} \Vert _{r}\lesssim2^{-j(s+1-\frac{2}{r})} $$

thanks to $f_{\ast}\in B^{s}_{r, q}$ and Lemma 1.1. Therefore

$$e_{2}^{\ast}\lesssim(\ln n)^{p-1} \sum _{j=j_{1}^{\ast}+1}^{j_{1}}\sum_{i=1}^{3}2^{j(p-2)} \sum_{k\in\wedge_{j}} \bigl\vert \beta^{i}_{j, k} \bigr\vert ^{p}\lesssim(\ln n)^{p-1}\sum _{j=j_{1}^{\ast}+1}^{j_{1}}2^{-j(sp-\frac{2p}{r}+2)}. $$

Combining this with the choice of $2^{j_{1}^{\ast}}\sim(\frac{n}{\ln n})^{\frac{\alpha}{s-\frac{2}{r}+\frac{2}{p}}}$, we observe that

$$e_{2}^{\ast}\lesssim(\ln n)^{p-1}2^{-j_{1}^{\ast}(sp-\frac {2p}{r}+2)} \lesssim(\ln n)^{p} \biggl(\frac{\ln n}{n} \biggr)^{\alpha p}. $$

This with (4.19) implies that (4.17) holds for $\theta \leq0$. Hence

$$T_{3}\lesssim(\ln n)^{p-1} \biggl(\frac{\ln n}{n} \biggr)^{\alpha p} $$

follows from (4.15)–(4.17).

Therefore, the desired conclusion can be concluded by (4.4)–(4.8) with $\ell=1,2,3$, which completes the proof. □
